# Meaning and coping orientation of bereaved parents: Individual and dyadic processes

**DOI:** 10.1371/journal.pone.0178861

**Published:** 2017-06-01

**Authors:** Sara Albuquerque, Asuman Buyukcan-Tetik, Margaret S. Stroebe, Henk A. W. Schut, Isabel Narciso, Marco Pereira, Catrin Finkenauer

**Affiliations:** 1 Cognitive and Behavioural Center for Research and Intervention, Faculty of Psychology and Educational Sciences, University of Coimbra, Coimbra, Portugal; 2 CICPSI, Faculty of Psychology, University of Lisbon, Lisbon, Portugal; 3 Psychology Program, Faculty of Arts and Social Sciences, Sabanci University, Istanbul, Turkey; 4 Department of Clinical Psychology, Utrecht University, Utrecht, The Netherlands; 5 Department of Experimental Psychopathology & Clinical Psychology, University of Groningen, Groningen, The Netherlands; 6 Utrecht University, Utrecht, The Netherlands; University of Louisville, UNITED STATES

## Abstract

The present study aimed to examine whether bereaved parents “meaning-made”–defined as results of attempts to reduce discrepancies between the meaning assigned to the death of the child and self and world-views—was influenced by their own and their partner’s coping orientations. Coping orientations were conceptualized within the Dual Process Model, which entails loss coping orientation (LO; focus on the loss itself), restoration coping orientations (RO; focus on stressors that come about as an indirect consequence of the bereavement), and a flexible oscillation between both coping orientations. The sample consisted of 227 couples identified through obituary notices in local and national newspapers, who provided data at 6, 13, and 20 months after the death of their child. At all three points of measurement, both partners independently completed the Dual Coping Inventory (DCI) and a scale developed by the authors about meaning-made from the loss. Data were analyzed using a multi-level Actor-Partner Interdependence Model. Results show that the combination of parents’ own LO and RO (operationalized through the interaction effect between LO and RO) have a positive effect in parents’ meaning-made. Partners’ LO have a negative effect in parents’ meaning-made. These results highlight the importance of, in the context of parental bereavement, being flexible by using both coping orientations, and of acknowledging the interdependence between partners, namely, the interpersonal process by which partner’s coping affect one’s meaning-made.

## Introduction

The death of a child can have a powerfully disrupting impact on parents’ world views [1] and clinical theorists and researchers have converged in emphasizing meaning-making as a crucial component of the adaptive adjustment process to bereavement [2, 3]. In this context, a question that gains particular relevance is who, and under what circumstances, is most likely to achieve “meaning-made” (an outcome-of-coping variable, defined by Park, 2010) after the loss. Because meaning-made requires energy and effort, personal resources, such as coping orientations (i.e., how people go about dealing with the loss of a loved one), may have an impact on the extent to which individuals engage in such processes and their eventual outcomes (e.g., meaning-made) [4]. This longitudinal study sought to examine the role of coping orientations in meaning-made after child loss.

Adjustment to the loss of a child is not only a matter of coping with individual grief, but also encompasses relational processes. Studies have shown that one partner’s coping affects the other partner’s adjustment [5, 6]. Research also showed that one partner’s support affects the other partner’s ability to make meaning from the loss [7–9]. Therefore, the partner’s coping orientations represent another important resource that may affect the meaning-making process. Adopting an *interpersonal* perspective that incorporates the examination of interdependence among bereaved couples, this study also sought to investigate whether, besides their own, partner’s coping orientations also impact on parents’ meaning-made.

In the following sections, we first conceptualize meaning-made, before providing an overview of studies on meaning-made among bereaved parents and its association with psychological and/or physical health outcomes. We then review intrapersonal studies on the association between coping strategies and meaning-made; finally, we review the evidence on the interpersonal impact of meaning-made, that is, how partners affect each other’s meaning-made.

### What constitutes meaning?

Meaning has received growing empirical and theoretical attention in the grief literature. Attempting to gain better understanding of bereavement and its consequences, recent models consider grieving as a process whereby people commonly direct coping efforts toward making sense of their lives, selves, and the world after a loss [2, 10]. Although researchers commonly recognize the importance of meaning in bereavement, there is less consensus on what constitutes meaning. In fact, the different uses and conceptualizations make it difficult to uniformly define meaning [11].

In an attempt to empirically operationalize and examine component parts of meaning, Park [11] engaged in an extensive and integrative review on this topic. This author distinguishes two forms of meaning: meaning-making and meaning-made. Authors have proposed that adaptive adjustment to stressful events involves reducing the discrepancy between the meaning attributed to the event and global beliefs about the world and self [12]. Meaning-making refers to the efforts that people engage to diminish this discrepancy, while meaning-made denotes the changes resulting from such processes. In the present study, we focus on meaning-made as proposed in Park’s [11] review, thus defined as changes derived from efforts to increase consistency between the meaning given to the event and global beliefs of the world and the self, that is, the attempts to reframe the loss so that it is less threatening to such beliefs.

These reframing efforts consist of both sense-making and benefit-finding [13]. Sense-making embodies the ability to achieve a subjective and benign understanding of the loss. Benefit-finding entails the capacity to identify benefits or to recognize a “silver lining” in the personal or social consequences of the loss (e.g., enhanced empathy, reordered life priorities and goals, a closer connection to other people) [3]. Although conceptually and theoretically different, benefit-finding and sense-making are similar in that they aim at reappraising the loss in a way that it is less threatening in terms of beliefs about the world and the self [3, 14]. Meaning-made, despite sounding contrived, since it reflects an outcome, is a process of continuous construction, as we discuss next, with meanings that can continuously be reconsidered or revised. Indeed, research has shown that meaning-made is not stable across time. For example, reporting meaning-made at approximately two-and-half months after spinal cord injury did not predict reporting meaning-made approximately five or thirteen months later [15]. Also, Cordova et al. [16] stated that the positive association of the meaning-made-related concept of posttraumatic growth with health outcomes may depend on the time that has elapsed since the trauma [16]. Finally, in their meta-analytic review Prati and Pietrantoni [17] reported that the study design (longitudinal vs. cross-sectional) significantly moderated the effect of positive reappraisal coping on growth. These findings justify the analysis of meaning-made after loss over time.

### Mental health consequences and meaning-made among bereaved parents

The death of a child can be particularly disruptive for parents’ basic beliefs of predictability, order, and justice in the world [1]. As children often imbue life with purpose and meaning [18], bereaved parents are left with views of the world that reflect meaninglessness, randomness, and uncontrollability [1]. The death of a child may seem utterly meaningless and parents may pose questions surrounding what their lives will be like and who they are without their child [3].

When a child dies, parents are deeply affected, both physically and psychologically [19]. The persistence of their suffering may vary as a function of their struggle to make meaning from their loss. In line with this, prior studies demonstrated that parents’ difficulties with making meaning often persist for extended periods of time, and for those who initiate a search for a meaning but made none, the risk for poor adjustment increases substantially [8, 20, 21]. Also, there is evidence of the beneficial effects of achieving meaning-made in bereaved parents, namely regarding their grief intensity [22] and their marital adjustment [8]. These relational beneficial effects of achieving meaning-made are particularly of valuable given the higher risk of marital dissolution in bereaved parents, in comparison to non-bereaved parents [23, 24]. These findings highlight the importance of focusing on the mechanisms underlying this process [25]. The current study focuses on one mechanism potentially contributing to meaning-made: coping processes.

### How is meaning found: The role of coping flexibility

Recent research has underlined the importance of flexibility in coping and emotion regulation, emphasizing adaptability [26, 27]. As an example, Bonanno and Burton [28] proposed the notion of regulatory flexibility, suggesting that flexible interchanging between emotion regulation strategies that are sensitive to changes in context may be more important than the global use of some strategies over others [28]. Accordingly, flexibility,—defined as the ability to enhance or suppress emotional expression in accordance with situational demands [29],–was found to be positively associated with posttraumatic growth [30], suggesting that meaning-made may be boosted by a combination of both emotion-oriented strategies and emotion-suppressing strategies.

This idea is congruent with the Dual Model Process of Coping (DPM) with bereavement developed by Stroebe and Schut [31], which highlights the need for a flexible oscillation between loss coping and restoration coping orientations, involving a regulatory process of both confrontation and avoidance. Loss coping orientation (LO) refers to addressing and working through aspects of the loss experience itself, and involves, for instance, crying, yearning, missing and remembering the lost person. Restoration coping orientation (RO), on the other hand, involves addressing the secondary stressors that come about as an indirect consequence of the bereavement (e.g., changing identity and role or mastering new skills) and the process of confronting and dealing with these as they occur in current, ongoing life.

The DPM’s specification of emotion regulation processes, particularly through the construct of oscillation, highlights the potential usefulness of this model in analyzing mechanisms underlying meaning-made. Research among bereaved parents has shown that the way parents cope affects their adjustment [5]. For example, using the DPM constructs, Wijngaards-de Meij et al. [6] reported that LO was predictive of higher levels of grief and depression, while RO was related to lower levels of grief and depression. However, to our knowledge, the link between the DPM constructs–loss and restoration coping orientations, and oscillation—and meaning-made, has not yet been empirically tested (the Wijngaards-de Meij et al. study did not operationalize oscillation). Given the DPM proposal that adaptive coping requires both confrontation and avoidance of loss and restoration stressors [31], we will consider loss and restoration coping orientations and, more importantly, the combination of both coping orientations as potentially contributing factors to meaning-made.

Relying on the adaptive contribution of both flexibility and the oscillation processes (proposed in the DPM), we expected that attention to both loss and restoration coping orientations (i.e., oscillation) will positively impact meaning-made of bereaved parents. Also, we propose an operationalization for oscillation. We predict that those bereaved parents who show no oscillation between LO and RO will adapt more poorly, showing lower levels of meaning-made. If there is oscillation, we predict higher levels of meaning-made.

### The relational context and individual adjustment

Parents within a couple share the loss of their child and are therefore confronted with their loss as an interdependent dyad [5]. Accordingly, when the couple loses a child, it seems plausible that parents are not only affected by their individual coping, but they are also affected by their partner’s coping. Yet, to our knowledge, studies have rarely considered this interpersonal aspect of losing a child. One exception is a study by Wijngaards-de Meij et al. [6] who examined the effect of the loss and restoration coping orientations of both bereaved parents on the adjustment process after the loss of their child. These authors found that the partner’s RO was conducive to better adjustment, especially for fathers. Specifically, for fathers, having a partner who had high RO was related to less depression and less severe grief intensity. For mothers, however, the partner’s coping was unrelated to their adjustment. In a further study of parents’ interpersonal coping processes, Stroebe et al. [5] showed that partner-oriented self-regulation (POSR), defined as the avoidance of talking about the loss and remaining strong in the partner’s presence with the intention to protect the partner, increased both partners’ grief intensity. Finally, a recent study has shown that joint dyadic coping (coping together and activating shared resources) helped the parents work through their grief as a couple and also individually [32]. Taken together, these findings underline the importance of examining interpersonal effects in couples who lost a child.

To our knowledge, no study has examined such interpersonal processes in relation to meaning-made. This is surprising, especially in light of the evidence showing that meaning-made processes are not purely private activities, but are pursued in the context of interpersonal relationships [2]. It has been suggested that achieving meaning-made following loss is negotiated in the social and family context [2, 33], and that the ability to construe meaning may depend on a supportive and validating social environment [2]. Also, studies have shown that partners’ support can serve as a basis for meaning-made in different loss contexts [34], including parental bereavement [7–9].

In light of these findings, it is important to consider individual coping orientations and meaning-made both at the individual and interpersonal level. Therefore, the present study adopts a dyadic perspective, focusing on how both partners’ loss and restoration coping orientations shape their meaning-made after losing a child. Given the evidence that meaning-made is a dynamic process that unfolds over time [15, 16], our study adopted a longitudinal design.

### The present study

The aim of the present study was to examine the role of flexibility between loss and restoration coping orientations on meaning-made among parents who had lost a child in a three wave, longitudinal study. Embracing a dyadic perspective and using a prospective, longitudinal design, our study aimed to assess whether:

- Parents’ meaning-made is influenced by their own coping, that is, the effect of a person’s coping (the predictor) on his/her own meaning-made (the outcome). We hypothesized that the combination of loss and restoration coping orientations would positively impact the meaning-made, more than loss and restoration on their own (Hypothesis 1).
- Parents’ meaning-made is influenced by their partners’ coping, that is, there is an effect of partner’s prior coping (the predictor) on a person’s own meaning-made (the outcome). We hypothesized that the combination of partner’s loss and restoration coping orientations would positively impact parents’ meaning-made, more than loss and restoration on their own (Hypothesis 2).

## Materials and methods

### Participants

Our dataset overlapped with the datasets of Wijngaards-de Meij et al. [6] and Stroebe et al. [5]. The sample consisted of 227 heterosexual couples who provided data at 6, 13, and 20 months after the death of the child (including young adult children, as long as these children had not started a family life of their own). Parents’ age ranged between 23 and 75 years (*M* = 40.72 years, *SD* = 9.54), 82.2% of the parents had not experienced child loss before, 96.9% were the biological parents, and 19.7% had no other children. Child’s age at the time of death ranged between stillborn and 30 years (*M* = 9.85 years, *SD* = 9.93). A total of 67.7% of the deceased children were boys. The causes of death varied greatly, including neonatal death, illness, accident, suicide, and homicide. Answers regarding the expectedness of death were given on a five-point scale (*M* = 1.86, *SD* = 1.35). Higher scores indicate greater expectedness.

### Procedure

The study was approved by the Research Institute of Psychology and Health’s ethical committee at Utrecht University, The Netherlands. In total, 463 couples who had lost their child were identified through obituary notices in local and national newspapers in the Netherlands. Single parents and bereaved parents who were grandparents (i.e., those parents whose deceased child was a parent him/herself) were excluded from this study, resulting in a final sample of 227 couples. Five and half months after the loss, parents were sent a letter and were called by phone to inquire about participating in the study. Written informed consent was obtained from all study participants prior to data collection. The design of the study was longitudinal, with data collections taking place at the three waves of sampling (6, 13 and 20 months post loss).

The non-participation percentage at 13 months and/or at 20 months post loss corresponds to 18.5% of the total participants. We found no differences between respondents and non-respondents at 13 months and/or at 20 months post loss regarding parents’ age [*F*(1, 442) = 1.44, *p* = .230], child’s age [*F*(1, 447) = 0.05, *p* = .816], meaning-made [*F*(1, 446) = 0.003, *p* = .959] and RO [*F*(1, 446) = 1.37, *p* = .242]. However, parents that did not participate at 13 months and/or at 20 months post loss reported lower LO than those who stayed in the study [*F*(1, 446) = 7.65, *p* = .006].

### Measures

Parents’ and children’s sociodemographic data (e.g., age, sex) as well as information regarding the circumstances of the death (e.g., type of death, time since death) were collected at the first assessment point after their loss. At all three points of measurement, both partners independently completed a set of questionnaires.

#### Coping orientations

Loss coping and restoration coping orientations were assessed using the Dual Coping Inventory (DCI), a measure developed for examination of these DPM parameters by Wijngaards-de Meij [35]. The DCI, which is theoretically based on the DPM, assesses two coping scales: loss coping orientation and restoration coping orientation. The subscale loss coping orientation (LO) consisted of three items: “I am occupied with the loss of my child”; “I dwell on my sorrow”; and “I think of our deceased child”. The subscale restoration coping orientation (RO) included four items: “I direct my thoughts toward the future”; “Despite everything, I am trying to make the best of it”; “I try to look ahead”; and “I am trying to go on with my life”. Answers are given on a five-point response scale, ranging from 1 = *not at all* to 5 = *very much*. In this study, the scores on the average of LO and RO subscales and on the operationalization of the oscillation/flexibility between these two orientations were used. Oscillation, as a dynamic process, is notoriously difficult to measure. Nevertheless, given the importance of the construct, we made an effort to test it, which resulted in the following operationalization: oscillation was operationalized as the combination of both LO and RO, therefore analyzed through an interaction between LO and RO. In this study the Cronbach’s alphas across waves ranged from .77 to .82 for LO and from .87 to .92 for RO. A confirmatory factor analysis (CFA) showed a good fit of the DCI in our sample: *χ*^2^(12) = 65.82, *p* < .001, comparative fit index (CFI) = 0.99, Tucker-Lewis index (TLI) = 0.97, and root mean square error of approximation (RMSEA) = 0.06 (90% CI 0.04–0.07).

#### Meaning-made

Meaning-made from the loss was assessed using a scale developed by the authors. This scale comprised five items, pertaining to sense-making (e.g., “I think about the loss in one way or another”, “I think the loss has a meaning that we do not know”), and to benefit-finding (e.g., “I also see good sides to the loss”, “I draw strength from the loss” and “Despite everything I try to derive something positive from it”). An exploratory factor analysis suggested a one-factor structure for the scale, accounting for 61.6% of the total variance. Answers are given on a five-point response scale, ranging from 1 = *rarely/never* to 5 = *very often*. The Cronbach’s alphas in this study ranged from .83 to .84 across waves.

### Data analysis

Pearson’s correlations between the study variables (based on the average of the variables for all three points of measurement) and correlations for comparison between men and women and across time were computed. All tests were conducted using a two-sided alpha level of 0.05. Analyses of variance (one-way ANOVAs) were used to analyze gender effects and time effects separately for women and men.

Given the non-independence of the members of a couple and the non-independence of the longitudinal measures within one partner, we used the Actor-Partner Interdependence Model (APIM) and multilevel modeling using SPSS 20.0’s Mixed Models [36, 37]. In the APIM, actor and partner effects are estimated simultaneously, while controlling for each other. In this study, the effect of a person’s prior coping (predictor) on his/her own meaning-made (outcome) is the actor effect, and the effect of partner’s prior coping on a person’s meaning-made is the partner effect. The need to use the APIM was confirmed by the calculation of the intraclass correlations (ICC) using variance components [37]. Regarding meaning-made, there were significant similarities between waves within the same individual (ICC = .73, *p* < .001) and between individuals within the same couple (ICC = .67, *p* < .001).

Regarding data structure, the data from the three waves were nested within individuals, and data from the two bereaved parents were nested within couples. Time since death was the lowest level (first level), the individual parent was the second level and the couple was the third level. As covariance type, we specified first order autoregressive structure [AR(1)], suggested for repeated measures models [37].

Multilevel analysis has advantages with respect to dealing with missing data, as it leads to unbiased estimates when the panel attrition (individuals who, after one or more measurement occasions, dropped out of the study) is assumed to follow a pattern defined as missing at random [38].

Using repeated cross-sectional associations, we first examined the APIM with main actor’s and partner’s LO and RO effects included, and the interactive effect between LO and RO of the actor (actor x actor interactions) and of the partner (partner x partner interactions). This model allows the examination of whether it is the combination of actor’s LO and RO and partner’s LO and RO that determines actor’s meaning. To examine whether there were longitudinal effects over the course of 14 months, we conducted residualized lagged analysis [39]. We tested the same model as above, but now used the previous LO and RO to predict following meaning-made (e.g., wave 1 variables to predict wave 2 meaning-made, wave 2 variables to predict wave 3 meaning-made), and controlling for the previous meaning-made. For the sake of transparency, we include findings from both the repeated cross-sectional associations and residualized lagged analysis. Nevertheless, we discuss the findings from the latter analysis in detail, given that it better reflects the regression model inherent to the study aims, that is, to explore the effect of own and partner’s coping orientations on one’s meaning-made.

In order to increase the interpretability of results, all predictors were standardized to the grand mean. We examined the form of these interactions by plotting predicted values one standard deviation above and below the means of the moderators [40]. Because meaning-made was a skewed variable, we conducted analyses including square-root transformations of meaning-made; the findings remained unchanged. All analyses were repeated controlling for gender, and the findings were practically the same.

## Results

### Descriptive analyses

The comparison of LO and RO scores of men and women revealed that men reported lower LO [*F*(1, 1233) = 137.84, *p* < .001] and higher RO [*F*(1, 1233) = 4.72, *p* = .030] than women. There were no gender differences regarding meaning-made [*F* (1, 1225) = 0.99, *p* = .320]. The ANOVA comparing the parents’ LO and RO scores of the three time assessment points (separately for men and women) revealed that women reported lower LO over time [*F*(2, 623) = 10.19, *p* < .001]. There were no differences regarding women’s RO over time [*F*(2, 629) = 0.70, *p* = .495] and regarding men’s RO [*F*(2, 600) = 1.03, *p* = .358] or LO [*F*(2, 600) = 1.14, *p* = .321] over time (see Table 1).

**Table 1:** Level of meaning-made and coping at the three time points.

		T1		T2		T3	
		*M*	*SD*	*M*	*SD*	*M*	*SD*
Meaning-made	Men	2.01	0.89	1.90	0.82	1.88	0.77
	Women	1.10	0.87	1.92	0.84	2.03	0.90
RO	Men	3.70	0.89	3.59	0.82	3.60	0.84
	Women	3.48	0.94	3.51	0.95	3.59	0.91
LO	Men	3.41	0.91	3.40	0.86	3.29	0.88
	Women	4.07	0.74	3.91	0.75	3.75	0.74

Note: T1 = 6 months; T2 = 13 months and T3 = 20 months

Regarding the Pearson’s correlations for the study variables (based on the average of the variables for all three points of measurement), meaning-made was negatively correlated with LO (*r* = -.06, *p* = .038) and positively correlated with RO (*r* = .24, *p* <. 001). RO and LO were negatively correlated (*r* = -.22, *p* < .001). The correlation between LO and RO was significantly higher among men, comparing to women (*Z* = 2.15, *p* = .032). There were no significant differences in the correlations across time.

### Actor-partner analyses

#### Parents’ coping orientations and parents meaning-made (actor effects)

Table 2 presents the results of the actor-partner analyses for both the cross sectional and the longitudinal model. To examine whether the combination of LO and RO positively impacted meaning-made (Hypothesis 1), the interaction effect between LO and RO of the actor (actor x actor effects) was calculated. The results from the repeated cross-sectional associations revealed that the interaction between LO and RO of the actor was not significant. A main effect for RO emerged, indicating a positive effect on meaning-made (*b* = 0.20, *SE* = 0.03, *p* < .001). Longitudinally, the interaction between LO and RO of the actor was significant (*b* = 0.07; *SE* = 0.02; *p* = .005). These results indicate that the combination of RO and LO was positively associated with meaning-made. No main effects for actor’s LO or RO emerged.

**Table 2:** Actor-partner effects in cross-sectional and longitudinal analyses.

	Estimate (*SE*)	*df*	*t*
**Cross-sectional model**			
A-LO	0.02 (0.03)	1071.24	0.76
A-RO	0.20 (0.03)***	1126.92	7.53
P-LO	-0.05 (0.03)*	1069.77	-2.02
P-RO	0.05 (0.03)	1136.33	1.88
A-LO X A-RO	0.01 (0.02)	1042.57	0.50
P-LO X P-RO	0.00 (0.02)	1019.60	0.10
**Longitudinal model**			
Meaning-made-E	0.72 (0.03)***	533.97	28.15
A-LO	-0.02 (0.03)	741.41	-0.97
A-RO	-0.04 (0.03)	749.49	-1.55
P-LO	-0.06 (0.03)*	728.45	-2.55
P-RO	-0.19 (0.03)	729.23	-0.77
A-LO X A-RO	0.07 (0.02)**	741.58	2.83
P-LO X P-RO	0.02 (0.02)	742.87	0.91

Note. SE = standard error; Meaning-made-E = earlier values of meaning-made; A-LO = actor loss orientation; A-RO = actor RO; P-LO = partner loss orientation; P-RO = partner RO.

* p < .05;

** p < .01;

*** p < .001.

### Simple slopes analysis

**Fig 1:**
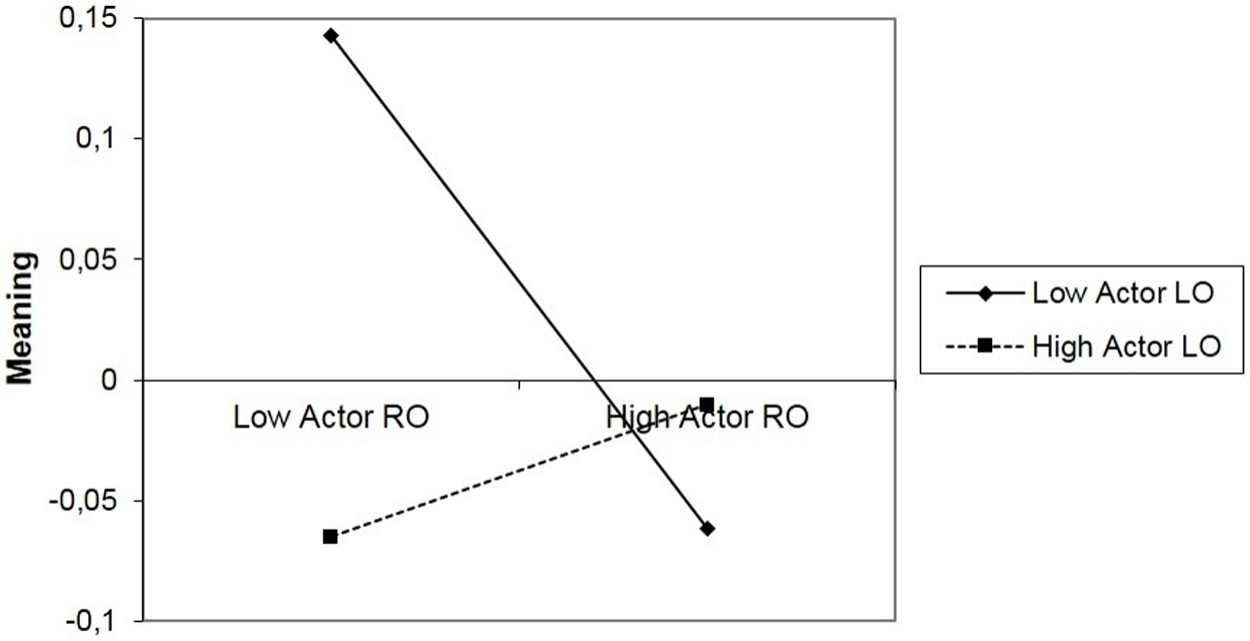
Interaction between loss orientation and restoration orientation of the actor (loss coping orientation as the moderator).

Given the significant actor’s LO or RO interaction, we conducted simple slopes analysis to examine whether the association between RO and meaning-made was different for individuals with high levels of LO in comparison with those with low levels of LO. These analyses revealed that at high levels of LO, there was no association between RO and meaning-made (*b* = 0.03; *SE* = 0.03; *p* = .430). At low levels of LO, however, there was a negative association between RO and meaning-made (*b* = -0.11; *SE* = 0.04; *p* = .004), indicating that for a parent who is low on LO, high RO was negatively related to meaning-made (Fig 1).

In addition, we examined whether the association between LO and meaning-made was different for parents with high levels of RO in comparison with those with low levels of RO. These results revealed that at high levels of RO, there was no association between RO and meaning-made (*b* = 0.04; *SE* = 0.03; *p* = .193). At low levels of RO, however, there was a negative association between LO and meaning-made (*b* = -0.09; *SE* = 0.04; *p* = .013), so for a parent who is low on RO, LO was negatively related to meaning-made.

In sum, taking into account the significant interaction effect, in support of our prediction, the results show that the combination of LO and RO predicted meaning-made after a child’s death. Also, these findings indicate that when parents were high in LO, their RO did not predict their meaning-made. Conversely, when parents were low in LO, their RO was negatively associated with their meaning-made. In the same way, when parents presented higher levels of RO, their LO did not predict their meaning-made. Conversely again, when parents were low on RO, their LO was negatively associated with their meaning-made. Lowest level of meaning was present when both LO and RO are high and in combination with each other.

Regarding partner effects (Hypothesis 2), partner’s LO had a negative effect on parents’ meaning-made cross-sectionally (*b* = -0.05, *SE* = 0.03, *p* = .044) and over time (*b* = -0.06, *SE* = 0.03, *p* = .011). The interaction between LO and RO of the partner (partner*partner interaction) and the partner’s RO effects were not significant neither cross-sectionally nor longitudinally, that is, partner’s RO and the combination of partner’s RO and LO were not associated with meaning-made.

### The effect of gender

Finally, we examined whether the actor and partner effects remained after controlling for gender. Cross-sectionally, the model showed a significant main effect of RO (*b* = -0.20, *SE* = 0.03, *p* < .001), and a marginal main effect of partner LO, (*b* = -0.05, *SE* = 0.03, *p* = .064). Longitudinally, the model also showed an interactive effect of LO and RO (*b* = 0.06, *SE* = 0.02, *p* = .006), and a marginally significant main effect of partner LO (*b* = -0.05, *SE* = 0.03, *p* = .063). When including gender in the model, the results remained the same. In other words, the patterns described above held controlling for the effect of gender.

## Discussion

In this study, we assessed whether parents’ meaning-made was influenced by their own and their partner’s coping orientation. As hypothesized, we found that longitudinally there was no effect of the coping orientations independently of each other, but there was a positive effect of the combination of LO and RO on meaning-made. This finding is consistent with studies showing the protective role of flexibility, in which this variable was found to be positively associated with posttraumatic growth [30] and with the postulation of the importance of oscillation, the need to confront both LO and RO stressors proposed by the DPM [31]. Regarding LO, research has shown the contribution of conscious and deliberate processing of trauma-related information and emotional states in growth [41]. However, when excessive and ongoing, this emotional processing may take a ruminative form, being intrusive, disruptive, and painful without being productive [42, 43]. Being rigidly loss-oriented, the person continually dwells on the painful aspects of the loss without getting any closer to finding a solution that attenuates their suffering and, possibly, to make a meaning for their loss. Therefore, conscientiously avoiding loss-related information by focusing on the present and future (RO) might be an important strategy to prevent being stuck in unproductive ruminations about the loss. In addition, our findings on the presence of the lowest level of meaning when both LO and RO were high (and in combination with each other), suggest that high levels of LO or RO may actually be detrimental to the bereaved person. These findings are compatible with the Stroebe and Schut [44] recent suggestion that a bereaved person may encounter more loss- and/or more restoration-oriented stressors than he or she feels able to deal with (i.e. overload), resulting, for example, in feeling overwhelmed.

We also found significant partner effects, which reinforces the importance of interpersonal effects in couples who lost a child, as found in other analyses of this data set [5, 6] and the role of close relationships in meaning-made between partners in a relationship. Specifically, we found that the partner’s LO coping had a negative effect on actor’s meaning-made. This finding seems consistent with the view that the ability to make meaning from the loss may be influenced by a supportive and validating social environment [2]. Indeed, by being more loss-oriented, the partner may be more focused on his/her individual loss and not be as open and available to support the other partner. Meaning-made is proposed to be potentially constructed and negotiated within the family and couple context [33]. It is possible that LO is associated with the unavailability of the partner, which may help explain the negative effect of a partner’s LO on one’s ability to make meaning from the loss. This notion is in line with research showing that when partners are in a negative mood, they are more self-focused and less able to provide social support, compared to when they are in a positive mood [45]. An alternative explanation may be that, assuming that partner’s LO would be correlated with partner’s higher grief, there could be a contagion effect of grief intensity, therefore leading to lower meaning-made in parents. Finally, one could also speculate that this negative effect of partner LO is due to partner LO, which may lead to actor LO (prospectively), that in turn may predict the actor meaning-made. It would be of value to explore these hypotheses in future studies.

This study is not without limitations. We only compared parents within the same culture. As theories and prescriptions for dealing with loss should be sensitive to different cultural contexts [46], in future research, it would be relevant to examine whether the same pattern of results emerges in different cultures. Furthermore, the dual coping and meaning-made measures, although presenting reliable properties in this study, have not been formally validated. Also, the measures may not be ideal indicators of the underlying constructs of interest in this study. For example, although we used a worthwhile operationalization of oscillation, the DCI does not enable its direct measurement. The interaction between LO and RO pertains to the combination of both coping orientations but does not reflect a dynamic process of switching between these two constructs, as reflecting in the oscillation concept in the DPM [31]. Also, the DCI does not assess a range of secondary stressors associated with the loss, which are involved in RO, focusing instead mainly on visualizing life ahead without one’s child. Similarly, the measure of meaning-made focuses mainly on benefit-finding and sense-making and does not capture other components of meaning-made (e.g., identity reconstruction; for a review see [11]). However, in contrast with the wide use of a single-item scale in meaning-made and meaning-making literature [11], we did use a broader and more complex scale intended to grasp the complexity of the meaning-made phenomenon. Nevertheless, the similarity and difference of our meaning-made measure compared to others should be examined in future studies. Finally, future studies should test whether our results hold across different causes of death and ages of the child.

Beyond these limitations, some major strengths of this study are also noteworthy. First, the study comprised methodological improvements compared with previous studies, namely it included a large sample size of bereaved parents, had a low attrition rate, and was longitudinal. Given the results of this study in future research, the measurement moments could usefully be more extended in time. This would also follow from previous studies in the literature showing that some sort of meaning-made can only be achieved several years after the death of a child [8]. Second, our study included both husbands and wives, which provided us with the opportunity to consider the interdependence between partners as well as dyadic effects and processes. Finally, as noted earlier, these results suggest that close relationships may play an important role in fostering meaning-made between partners in a relationship. Our study is the first to outline the interpersonal process by which own and partner’s coping affect one’s meaning-made.

On a clinical level, if replicated, our findings suggest the need to target–but only where treatment is indicated/appropriate [47]–both partners and incorporate interpersonal components into intervention programs for parents coping with the loss of a child. Fostering reconstruction of a world of meaning would seem of major therapeutic importance [47], particularly given the evidence of the beneficial effects of achieving meaning-made for the reports of grief intensity and marital satisfaction [8, 22].

Our results support the view that parents’ interpersonal experience should be taken into account, while at the same time recognizing that reactions are inextricably tied to the bereaved person´s intrapersonal feelings of pain and loss. In other words, our findings suggest the importance of focusing on the impact of the partner’s coping on meaning-made, in addition to that of the bereaved person´s own coping orientations. For example, including information on the possible detrimental effects of partner’s LO and finding strategies to limit it could be of particular value. Finally, we highlight the need to explore meaning-made beyond first causal attributions or benefit finding, given the evidence that the meanings-made are continuously revisited and altered [47].

In future research, the potential processes by which a partner’s coping can influence a person’s meaning-made could be usefully investigated. Also, the examination of the effect of own and partner’s coping orientations for the dimensions of meaning-made (benefit-finding and sense-making) separately would be important. Research has shown that although related, these components may differ; for example, sense-making is more likely than benefit-finding to be a finite process [43]. In addition, besides separate analyses, these components could also be analyzed at different time points. The findings of Davis et al. [15] suggest that making sense of the death of a loved one is an adaptive process within the first year following the death, while benefit-finding is adaptive after the first year. Also, despite the existence of meaning-made, a better understanding of the quality and content of the meaning-making attempts and the meanings found is also important. Furthermore, future studies could implement a mixed method design, providing quantitative and qualitative data on the mechanisms by which coping orientations contribute to meaning-made. Also, information on how partners communicate (verbally and non-verbally) their coping orientation to each other, for example, examining which behavior (or lack of behavior) drives the effects, could also be valuable. Finally, considering the differences in our cross-sectional and longitudinal findings, future research should give priority to longitudinal studies but also compare them with cross-sectional findings.
